# Experiences of Professional Helping Relations by Persons with Co-occurring Mental Health and Substance Use Disorders

**DOI:** 10.1007/s11469-017-9780-9

**Published:** 2017-06-21

**Authors:** E. Brekke, L. Lien, S. Biong

**Affiliations:** 1 Norwegian National Advisory Unit on Concurrent Substance Abuse and Mental Health Disorders, Innlandet Hospital Trust, Ottestad, Norway; 2 Faculty of Health Sciences, University College of Southeast Norway, Drammen, Norway; 3 NROP, Postal box 104, 2318 Brumunddal, Norway; 4 Faculty of Public Health, Hedmark University of applied sciences, Elverum, Norway

**Keywords:** Co-occurring disorders, Drug abuse, Mental disorders, First-person perspectives, Therapeutic alliance, Helpful relationships

## Abstract

Recovery in co-occurring mental health and substance use disorders often involves relationships with professional helpers, yet little is known about how these are experienced by service users. The aim of this study was to explore and describe behaviour and attributes of professional helpers that support recovery, as experienced by persons with co-occurring disorders. Within a collaborative approach, in-depth individual interviews with eight persons with lived experience of co-occurring disorders were analysed using systematic text condensation. The analysis yielded four categories of recovery-supporting behaviour and attributes of professional helpers and the ability to build trust cuts across all of them: Building trust through (a) hopefulness and loving concern, (b) commitment, (c) direct honesty and expectation and (d) action and courage. Services should allow for flexibility and continuity, and training should recognise the importance of establishing trust in order to reach out to this group.

Recovery in co-occurring mental health and substance use disorders (co-occurring disorders) often involves relationships with professional helpers, yet little is known about how these relationships are experienced by service users.

People with co-occurring disorders are considered hard to reach and retain in treatment (Padgett et al. 2008). While the prevalence of co-occurrence is well established (Landheim et al. 2006; Regier et al. 1990) and recommendations for treatment exist (Mueser and Gingerich 2013), services may still not match the needs of individuals with co-occurring disorders. This group is exposed to homelessness, poverty and unemployment, which may exacerbate symptoms and prevent recovery (Laudet et al. 2000; Margolese et al. 2004; Tsai et al. 2010). Persons with substance use problems face negative stereotypes, putting them at risk for discrimination (Bye et al. 2014). Many experience loneliness and a lack of belonging in mainstream society. These may be difficulties so unfamiliar to professional helpers that they fail to recognise them, let alone their impact on people’s lives. People with co-occurring disorders may prefer to ask peers for advice on health issues rather than professional helpers, because they believe professionals would not understand their life situation (Ness et al. 2014). This suggests that exploring service users’ experiences may be particularly relevant in order to improve services for this group.

Even if co-occurring disorders are associated with several life difficulties, there is hope for recovery in the long term (Drake et al. 2006). Originating among persons with lived experience, an understanding of recovery as a personal and social process that exceeds symptom reduction has gained foothold within the fields of mental health and substance use, adding to the traditional psychiatric understanding of recovery as ‘returning to normal’—symptom reduction that can be observed and rated by an expert (Anthony 1993; Deegan 1996; Laudet et al. 2009; Slade et al. 2012). Different definitions of recovery exist related to mental health and substance use. This lack of consensus may hinder clinical practice and research (Laudet 2007), but it may also acknowledge the fact that recovery means different things to different people. One distinction has been made between recovery *from* mental illness, indicating cure, versus recovery *in* mental illness, indicating enhanced quality of life regardless of cure (Davidson and Roe 2007). Further, one might differentiate between recovery as something that happens to and within an individual, and recovery as a social process that involves the larger community, and where the person is an active participant (Topor et al. 2011). Even if there is no clear consensus of the definition of recovery, there is an increasing agreement that multi-stakeholder definitions are valuable, which is also reflected in research (Neale et al. 2016).

Underpinning this study is an understanding of recovery as a personal and social process, which involves both recovery from and recovery in co-occurring disorders. Seeing recovery as a social process involves recognising everyday life as the central arena for change (Borg and Davidson 2008) while acknowledging structural factors and underlying social-psychological dynamics (Best et al. 2016). Recovery is understood as ‘a process of restoring a meaningful sense of belonging to one’s community and a positive sense of identity apart from one’s condition while rebuilding a life despite or within the limitations imposed by that condition’ (Davidson et al. 2007). In a previous study based on the material in this paper, participants described recovery as feeling useful and accepted, coming to love oneself, mastering life and emerging as a person (Brekke et al. 2017).

Previous studies that have examined the experiences of persons with co-occurring disorders with professional help report that service users appreciate that professionals are resource-focused, collaborating, flexible and accepting (Biong and Soggiu 2015), carry hope, promote mutual honesty and continuity of contact, are qualified to address both substance use and mental health conditions (Cruce et al. 2012) and show unexpected acts of kindness, in contrast to routinized encounters that may be experienced as dehumanising (Padgett et al. 2008).

Therapeutic alliance, which may be defined broadly as the collaborative and affective bond between therapist and patient, is established as a predictor of outcome in psychotherapy (Martin et al. 2000; Norcross and Wampold 2011). Relational factors also seem to be significant in other helping relations, such as community health interventions (Kidd et al. 2017; Ljungberg et al. 2015). Relational factors are insufficiently understood, operationalized and emphasised in research on mental health and substance use treatment (Davidson and Chan 2014; Miller and Moyers 2015). There is a need for research that explores therapist behaviour and qualities that enhance positive change, from the patients’ perspective (Norcross and Wampold 2011). Research on how professional helpers contribute to recovery has typically investigated mental health and substance use separately (Borg and Kristiansen 2004; Ljungberg et al. 2015). Further exploration of the perspective of persons with co-occurring disorders on how professional helpers may support recovery is needed (Cruce et al. 2012).

The aim of this study is to explore and describe behaviour and attributes of professional helpers that support recovery, as experienced by persons with co-occurring disorders.

## Materials and Methods

### Context

This study is part of a research project that investigates the recovery orientation of community mental health and addiction services in a local authority area in Eastern Norway. The community consists of agricultural areas, forested areas and two community centres (<6500 inhabitants). Drawing on literature on collaborative research (Moltu et al. 2013), an advisory group of six persons from the community has assisted the authors throughout the research process. They are two persons with lived experience of co-occurring disorders, one family member of a person with co-occurring disorders, and three professional helpers. The group has participated in developing the interview guide, the inclusion criteria and the recruitment strategy. They have been consulted in the data analysis for validation and for understanding the results in relation to the local context.

### Recruitment

A sampling strategy that aimed for diversity in age, gender, duration of contact with services, substance use and mental health problems was applied. Flyers were handed out by the staff of the local mental health and addictions team, at a peer support house, in the local narcotics anonymous group and at a low-threshold meeting place that provides harm-reduction health services for persons with substance use problems in the nearest town. Participants were able to join by e-mail or SMS, or by agreeing that staff members forward their telephone number to study personnel.

### Participants

The participants were four women and four men (see Table 1). All were in contact with the community health and social services at the time of the interview. Duration of contact with services ranged from 1 year to more than 10 years. They acknowledged that substance use and mental health problems seriously affected their everyday life, currently or in the past. They reported having used or using alcohol, amphetamines, benzodiazepines, opioids and/or cannabis. Most participants reported having used several substances. Four persons reported not using substances at the moment, one was in maintenance treatment and three persons were currently using substances at the time of the interview. The participants reported experiencing or having experienced affective disorder, anxiety, post-traumatic disorder, psychotic illness and/or hyperactivity disorder.

**Table 1:** Participants

Participant	Gender	Age	Maintenance	Occupation	Housing	Civil status
1	Man	75	Disability pension	None	Own house	Single
2	Woman	55	Disability pension	None	Rented	Cohab
3	Man	40	Disability pension	Student	Rented	Cohab
4	Woman	26	Social welfare	Job seeker	Rented	Single
5	Woman	54	Disability pension	None	Rented	Cohab
6	Man	54	Social welfare	Student	No fixed abode	Single
7	Man	52	Disability pension	None	Own house	Married
8	Woman	62	Disability pension	None	Rented	Single

### Data Collection

Eight semi-structured, in-depth individual interviews (Kvale and Brinkmann 2009) were carried out by the first author. An interview guide consisting of open-ended questions about what recovery means and what may lead to recovery was developed in collaboration with the advisory group. Concrete and detailed descriptions of behaviour and attributes of professional helpers that support recovery were sought. Participants were asked to describe their own personal experiences of encounters with professional helpers. Follow-up questions were asked, such as: ‘What was that like for you?’ and ‘How did that feel?’ Interviews lasted from 45 to 80 min.

### Analysis

Interviews were audiotaped and transcribed verbatim by the first author. Data analysis was guided by systematic text condensation (STC) (Malterud 2012) within a phenomenological approach (Giorgi 2009). STC is a descriptive and explorative method that aims at thematic analysis of meaning and content across cases. It offers prescriptive details for analysis which enable a process of intersubjectivity, reflexivity and feasibility, while maintaining transparency. Specific to STC is the procedure of incorporating text from all meaning units into an artificial quotation (step 4 below), safeguarding a systematic review of all meaning units in the material. Selective bracketing of the researcher’s pre-understanding was sought in the analysis process. Initially, all transcripts were read as a whole in order to gain an overall impression, resulting in preliminary themes. Secondly, the transcripts were systematically reviewed line by line, identifying, classifying and sorting meaning units into code groups. Thirdly, meaning units within each code group were sorted into subgroups. At the fourth step, all meaning units within each subgroup were reduced into an artificial quotation maintaining, as far as possible, the original terminology used by the participants. An authentic illustrative quotation was identified for each subgroup. Finally, analytic texts were developed, synthesising the contents of the artificial quotations and developing descriptions. The analytic texts were validated by returning to the full transcripts and asking whether our synthesis still reflected the original context. At steps 3–5, the advisory group was consulted, providing an understanding of the material from within the local context. The ‘Results’ section consists of analytic texts with supporting original quotes from the participants. Interviews were conducted, transcribed, and analysed in Norwegian. Analytic texts and supporting quotes were translated into English by the first author. The translated text was sent to a professional translator along with the original quotes in Norwegian. The names used in the quotes are fictional. The NVivo 10 software was used in the analysis.

### Ethical Considerations

The study was approved by the Norwegian Centre for Research Data (case no. 42244). Informed consent was a requirement for participation. Debriefing was integrated into the interview situation. Participants were offered the opportunity to get in touch with the first author after the interview. Details that could identify participants were removed before the material was shown to the advisory group. The members of the advisory group signed a declaration of confidentiality.

## Results

The analysis yielded four categories of recovery-supporting behaviour and attributes of professional helpers and the ability to build trust cuts across all of them: Building trust through (a) hopefulness and loving concern, (b) commitment, (c) direct honesty and expectation, and (d) action and courage.

### Building Trust through Hopefulness and Loving Concern

Participants appreciated professionals expressing faith in their possibilities for a better life. Experiencing that professionals believed in them was associated with reclaiming hope and starting to believe that positive change was possible.

Carl was at my house yesterday, and he told me that he believes in me. He said he’s got no doubt that I’ll make it. And it’s like, then I don’t doubt that, either.

Loving concern was described as a certain demeanour or presence which communicated respect, acceptance, concern and a fundamental goodness. A lack of disdainful attitudes, distance, moralism and arrogance further described this phenomenon. To listen carefully, to be interested in the other and to respect the other’s opinions, were mentioned as expressions of loving concern. Experiencing loving concern was related to feeling secure and trusting that the other wants the best for you. Several participants described their relationship to a professional as a ‘good match’, which could not be obtained with everyone. A sense of humour, warmth and a comfortable and non-authoritarian manner were mentioned as attributes that allowed for a good match.

You can talk to him about everything. (…) He knows more or less everything about me. And I trust that he wants the best for me.

Loving concern was experienced when professionals seemed sure of themselves, were conscious of their own role and did not bring their personal needs into the relationship. Several participants mentioned that professionals who seemed to be in harmony with themselves had treated them in a way that allowed them to regain a sense of dignity. Some described it as helpful when professionals with lived experience shared their experiences.

It’s how they receive you. You don’t feel like a patient (…) Well, we’re not equal, because they’re well, and I’m ill. But we’re equal all the same. Yes. It’s like, as they’re so self-confident, they actually make me feel well, too. You see.

A lack of hopefulness and loving concern was experienced when professionals acted negligently, too familiarly, not doing their job properly, or using the relationship to fulfil their own needs. One participant had found that a professional had seemed to hold her back when she was actually getting better, and wondered if it was done in order to keep helping her.

Like, their ego gets in the way of helping, they kind of become a person who needs to be seen by me. (…) Like, they’re unprofessional, they start mixing things. And then they start telling me things. And they don’t pay attention. (…) I think there are lots [of professionals] who try to be kind of a buddy and a friend and … it’s kind of unprofessional.

### Building Trust through Commitment

A continuous, long-term relationship with a professional was described as supporting recovery. Knowing each other well led to mutual trust and honesty, possibly preventing relapse into substance use. All participants appreciated that professionals spent time with them and some wished that professionals had more time.

When I meet Anna, she knows me so well. Some simple words from her, and just seeing her, and I can relax. (…) And she’s been worrying about me when she hasn’t seen me for a while. ‘Oh, there you are! Oh, I was so afraid that something might have happened to you’. Because she’s known me for so long.

Professionals insisting on making contact and not accepting cancellations was described as supporting recovery by some participants. This enabled participants to trust that the professional was interested in helping them and would not let them down. Some wished that professionals had been more insistent in the past.

It’s really important that they actually pester you a little bit. And that they think a bit about how to say things. Not: ‘Should I come and pick you up?’, if you have an appointment, but: ‘I’ll pick you up at twelve’.

The participants valued professionals that handled ups and downs and stood by them through relapses and times of mental distress. One woman described how a professional had not given up when change had seemed unlikely. The day she was ready to make changes, they were able to plan treatment with good timing. One person explained that a professional had been the only person he had seen for long periods of time and doubted that he would have managed without her. Illustrating a lack of commitment, another participant described how professionals seemed to distance themselves when his mental distress got worse, wishing they would instead approach him more during those periods.

I love Eric. But I’d like him to take me more seriously when I tell him that I’m struggling. Because when he realises that I’m struggling, he walks away. So you get punished twice, in a way. And when I’m doing well, he gets very happy. And it’s almost enough to push me into the ditch, you know. Because then he should come even closer instead of disappearing. Because he shies away, and thinks something is wrong. But I don’t know how to tell him this.

Keeping in touch with a local council employee during inpatient addiction treatment was appreciated. Close follow-up from a professional who knows you and whom you trust was helpful upon discharge from inpatient treatment. Making long-term plans for housing, work and finances was highlighted in this connection.

It’s when you get out (from treatment). The first year. That’s the toughest. And then it’s so important that this and that’s in place. It’s important to have support staff out there then, who understand you and who know who you are. (…) But to get there, there has to be cooperation while you’re in treatment, and build trust in each other and be honest with each other. That’s by far the most important thing. Because then I dare to call Sara and tell her how things are. But if I didn’t trust her, I would never have called. That’s the difference between hitting the floor and managing to recover.

### Building Trust through Direct Honesty and Expectations

The participants described it as helpful that professionals spoke their mind frankly and expressed concern about the participants’ current and future health situation, combined with advice for change, especially when the situation was serious. This had enabled participants to understand the severity of their situation and the need for change, even if it had felt painful at the time. Also, direct honesty made it easier to trust professionals without wondering if they had a hidden agenda.

I trust Hans. (…) He’s honest through and through. He doesn’t hide anything. And that doesn’t bother me, it’s just fine. It’s a lot better to have someone who calls a spade a spade, and no more fuss about it.

The participants appreciated if professionals were not easily manipulated and were skilled in addressing substance use. Several participants described a sense of empowerment when professionals made them understand, in a respectful way, that they were responsible for their own life. Some had experienced pity and ‘pampering’from professionals as unhelpful.

Many staff are used to treating suffering people in a certain way. They think they’re so-called ‘nice’, you know. (…) You can almost smell it when you enter a room with people like that. Whether I was drunk or sober, I would always realise who I was dealing with, who I could manipulate and play on their emotions, you know. You get to be a real expert at that.

It was appreciated by the participants when professionals followed up closely and expected efforts from them. Some had experienced routine checks, such as urine samples, as helpful. Some had found professionals to be afraid of being direct, which was not appreciated. One person had found that professionals expected too little from her after she was diagnosed with a severe mental illness, making it difficult for her to recover. Some had felt that professionals with lived experience were more honest and direct than others.

When I’m all hyper and distressed, people often think I’m on drugs. (…) But if one of the staff checks on me and does regular urine samples ... (…) It has to do with pride, too, to be allowed to show them. Because even if I was clean, I just cried all the time and everyone thought that I was high. But it’s better to get a hold on yourself and be able to show them: ‘I’m clean, you’re wrong’, kind of.

Several participants said that professionals should intervene to help children whose parents have mental health and substance use problems. Some participants felt that professional helpers should have intervened sooner to help their own children.

So I wonder: how’s it possible for the support services to watch a mother raving about in the streets, blind drunk, and then the child still lives with that mother. How the heck is it possible? This went on for several years in my case. (…) And I had contact with them, they even came to my house. And they didn’t do anything until the child started school.

### Building Trust through Action and Courage

Some mentioned that their mental health condition made it difficult to take part in valued activities, such as hiking, sports or socialising. The participants appreciated professionals urging them to be more active and accompanying them to activities, at least initially. Having a partner in their everyday activities had enabled participants to learn new skills, gain confidence and escape loneliness. Some participants wished that professionals would focus more on action in addition to talking.

You need to accompany people to the activities they can use as they want. Not just say: ‘Go there and do that’. I’ve experienced this myself. You know that it’s there, but you can’t manage to do it.

Acknowledging different aspects of their life situation, working hard to find out how to help, taking risks and having the courage to do more than just what is expected were all qualities appreciated in the staff. Professionals who were easy to get in touch with, and who said yes when asked for help, made it possible to ask for help without fear of being rejected. Helping out with practical, everyday issues and acting as a link to health and social services was appreciated. One participant had borrowed a trailer from a professional when he was moving, and found this to be a demonstration of trust which made him feel appreciated, hopeful and confident. Another participant described how professionals worked overtime in order to get her into acute treatment when she experienced a crisis. She now saw this as a crucial turning point in her recovery, expressing gratitude towards the professionals involved.

Liv has helped me with practical things, or other things. You know, made my life quite a lot easier. (…) I could just call, and she’d drive me to the supermarket, or to the doctor, or … yes. So she’s someone I trust.

Noticing actual changes in everyday life, such as improved health or solutions to financial difficulties, was described as very motivating. Some stated that they needed more help with sorting out financial problems, as they lacked skills in doing this. One woman said that the debt collectors would not listen to her when she asked for a payment plan on her debts. She thought that if a professional called in her place, they would trust him more and be willing to discuss solutions. Some of the participants also had close family members with mental health and substance use conditions, and would have liked more support from professionals in dealing with the burden of being a carer.

You have to do the job yourself, of course, but you need just that little bit of help. Not that much, really. So that you can kind of see: ‘Wow, it works!’, you know.

## Discussion

The present study adds to existing literature by providing descriptions of how trust can be established and maintained in helping relations with persons who live with co-occurring disorders. Hopefulness and loving concern, commitment, direct honesty and expectation and action and courage appeared as ways of establishing trust. Results from the present study support the argument that trust is a basic prerequisite for a therapeutic alliance through which other interventions may be delivered (Davidson and Chan 2014; Topor and Denhov 2015).

Hopefulness and loving concern resonate with elements of common factors in psychotherapy research which explain the effectiveness of therapeutic interventions beyond the specific effect of particular interventions. A comprehensive account of common factors is beyond the scope of this paper, but they particularly resonate with therapist qualities that facilitate a working alliance (Wampold and Imel 2015), such as empathy (Elliott et al. 2011) and positive regard (Farber and Doolin 2011). The theme ‘loving concern’ also resembles ‘acts of kindness’ (Padgett et al. 2008), ‘human warmth’ (Laugharne et al. 2012) and ‘shared humanness’ (Ljungberg et al. 2015) which appear from first-person accounts of helpful relationships within co-occurring disorders and severe mental illness. These seem to go beyond a mere professional relationship. However, unprofessional behaviour such as role confusion, loss of focus, or sloppiness, was described by participants as unhelpful. Trust has been described as a characteristic of hope inspiring relations in substance use counselling (Koehn and Cutcliffe 2012; Sælør et al. 2015), as well as an ecological aspect of settings that promote hope (Jason et al. 2016). In the present study, there seemed to be a reciprocal relationship between trust and hope, where the ability to communicate hope also enables trust.

A lack of trust in the system has been described by others, but was less explicit in the present study. A ‘threshold of trust’, which has to be trespassed in order to access services, has been described in low-threshold services for persons with co-occurring disorders (Edland-Gryt and Skatvedt 2013). It is probable that the design and recruitment strategy of the current study fails to access persons who currently do not trust the system. Most participants were middle-aged, and many had been in contact with different services for many years. Also, the focus on what leads to recovery may have resulted in less attention on negative experiences. Still, the fact that trust was highlighted by participants may suggest that they had experienced a lack of trust in the system in the past.

Professionals who were able to recognise the life circumstances of the participants and take action in order to solve practical problems and reduce loneliness were described as supporting to recovery in this study. This was different from disempowering people by doing things for them that they could very well do themselves. On the contrary, showing expectations and stressing responsibility were described as promoting recovery. Further, the lack of a patronising attitude was underlined as fundamental by many participants. The concept of *responsibility attribution* in helping relations may shed light on these nuances (Brickman et al. 1982). This theory suggests that models for help that attribute responsibility for solving the problem to the individual, without blaming him or her for the origin of the problem, are able to help eliminate deprivation, and still consider individuals as responsible and competent in helping themselves once the deprivation is gone. The phenomenon of building trust through action and courage described in this study may be understood as a manifestation of such a model of support.

Direct honesty and expectations from professional helpers should not be interpreted as confrontation, hostility or devaluation. A confrontational style has consistently been found to be ineffective in addiction therapy (Norcross and Wampold 2011). Direct honesty and expectations were perceived as helpful when they occurred within an atmosphere of hopefulness and loving concern, and the participants trusted that the professional helper wanted their best. This may indicate that direct honesty is easier to accept once a trusting relationship is established, but participants also described positive experiences of direct honesty in their first encounters with a professional helper. Direct honesty was particularly related to the professional helpers’ competence in understanding and addressing substance use. Similarly, in a study of preferred therapist characteristics by women who had been treated for anorexia nervosa, participants appreciated therapists’ ability to address anorexia in a straightforward and competent way (Gulliksen et al. 2012). Direct honesty may possibly be of particular importance within the cultural context of the present study: a rural area where the ability to use common, everyday language and appear unpretentious and down-to-earth will generally be perceived as trustworthy. This seems related to ‘establishing credibility by being genuine and honest’, which was described in a Canadian study of how counsellors inspire hope in persons with substance use problems (Koehn and Cutcliffe 2012).

The way services are organised influences professional helpers’ ability to engage in behaviour that builds trust. Systems that provide services for persons with co-occurring conditions need to recognise the importance of building trust or they risk making themselves inaccessible to the people they are meant to help. Service designs that allow for continuity, flexibility and engagement in everyday life may encourage the building of trust, but further research is needed.

### Limitations and Strengths

The methods do not allow for an immediate generalisation of the results, but rather an enhanced understanding of the phenomenon which may be transferred to other contexts. All participants lived in the same local area, and contact with professional helpers may be experienced differently in other contexts. The data contains experiences with professional helpers from different professions and different contexts, although the main focus is on the local addiction team. Participants seemed to value the same attributes in professional helpers across professions and levels within the health care system. However, a study which explicitly asked for valued attributes of professional helpers in other contexts, such as psychotherapy, would possibly elicit other descriptions.

The analytic approach in this study has a limited capacity for exploring processes over time (Malterud 2012). Qualitative studies with a narrative approach could shed light on how trust develops throughout the process of establishing a therapeutic relationship.

Diagnostic interviews were not conducted, and the description of participants relied on self-report in reply to general questions about mental health, substance use and life situation. This may be a limitation to the transferability of the results, since the symptom load was not accessed. Also, the problems described by the participants are varied, and one might question whether it is right to treat their experiences as representing the same phenomena. We believe that the participants’ descriptions are detailed enough to make judgements of relevance to other contexts, an important point being that all participants found that co-occurring conditions strongly affected their everyday life, either now or in the past.

## Conclusion

The study provides an enhanced understanding of how individuals with co-occurring disorder may experience relationships with professional helpers. Results suggest that building trust is fundamental in order to support recovery for this group. Professional helpers may establish trust through hopefulness and loving concern, commitment, direct honesty and expectations and action and courage. The ability to take action to solve practical problems while recognising people’s competence and responsibility for helping themselves appears as central. Services should be organised so that they allow for flexibility and continuity, and training should recognise the importance of establishing trust in order to reach out to this group. There is a need for further research on how professional helpers and services support or hinder recovery for persons with co-occurring conditions.
